# VHSV Single Amino Acid Polymorphisms (SAPs) Associated With Virulence in Rainbow Trout

**DOI:** 10.3389/fmicb.2020.01984

**Published:** 2020-08-27

**Authors:** Valentina Panzarin, Argelia Cuenca, Michele Gastaldelli, Anna L. F. Alencar, Francesco Pascoli, Thierry Morin, Yannick Blanchard, Joëlle Cabon, Lénaïg Louboutin, David Ryder, Miriam Abbadi, Anna Toffan, Carlos P. Dopazo, Stéphane Biacchesi, Michel Brémont, Niels J. Olesen

**Affiliations:** ^1^Division of Comparative Biomedical Sciences, Istituto Zooprofilattico Sperimentale delle Venezie (IZSVe), Padua, Italy; ^2^Unit for Fish and Shellfish Diseases, EURL for Fish and Crustacean Diseases, National Institute of Aquatic Resources, Technical University of Denmark (DTU), Kongens Lyngby, Denmark; ^3^Unit of Viral Diseases in Fish, Laboratory of Ploufragan-Plouzané-Niort, French Agency for Food, Environmental and Occupational Health & Safety (ANSES), Plouzané, France; ^4^Unit of Viral Genetics and Biosafety, Laboratory of Ploufragan-Plouzané-Niort, French Agency for Food, Environmental and Occupational Health & Safety (ANSES), Ploufragan, France; ^5^International Centre of Excellence for Aquatic Animal Health, CEFAS Weymouth Laboratory, Weymouth, United Kingdom; ^6^Departamento de Microbiología y Parasitología, Instituto de Acuicultura, Universidade de Santiago de Compostela, Santiago de Compostela, Spain; ^7^Virologie et Immunologie Moléculaires, Université Paris-Saclay, Institut National de Recherche pour l’Agriculture, l’Alimentation et l’Environnement (INRAE), Université de Versailles Saint-Quentin-en-Yvelines, Jouy-en-Josas, France

**Keywords:** VHSV, molecular markers, single amino acid polymorphism (SAP), virulence, rainbow trout

## Abstract

The Viral Hemorrhagic Septicemia Virus (VHSV) is an OIE notifiable pathogen widespread in the Northern Hemisphere that encompasses four genotypes and nine subtypes. In Europe, subtype Ia impairs predominantly the rainbow trout industry causing severe rates of mortality, while other VHSV genotypes and subtypes affect a number of marine and freshwater species, both farmed and wild. VHSV has repeatedly proved to be able to jump to rainbow trout from the marine reservoir, causing mortality episodes. The molecular mechanisms regulating VHSV virulence and host tropism are not fully understood, mainly due to the scarce availability of complete genome sequences and information on the virulence phenotype. With the scope of identifying *in silico* molecular markers for VHSV virulence, we generated an extensive dataset of 55 viral genomes and related mortality data obtained from rainbow trout experimental challenges. Using statistical association analyses that combined genetic and mortality data, we found 38 single amino acid polymorphisms scattered throughout the complete coding regions of the viral genome that were putatively involved in virulence of VHSV in trout. Specific amino acid signatures were recognized as being associated with either low or high virulence phenotypes. The phylogenetic analysis of VHSV coding regions supported the evolution toward greater virulence in rainbow trout within subtype Ia, and identified several other subtypes which may be prone to be virulent for this species. This study sheds light on the molecular basis for VHSV virulence, and provides an extensive list of putative virulence markers for their subsequent validation.

## Introduction

Viral Hemorrhagic Septicemia Virus (VHSV), member of genus *Novirhabdovirus*, species *Piscine novirhabdovirus*, possesses a bullet-shaped enveloped conformation and a negative-sense single-stranded RNA genome typical of the *Rhabdoviridae* family (Walker et al., 2018). Genes in the order 3′-N-P-M-G-NV-L-5′ encode five structural proteins with conserved functions among *Rhabdoviruses* (Schütze et al., 1999; Dietzgen et al., 2017). The nucleoprotein (N) is the major structural protein that tightly encapsidates the viral RNA. Together with its cofactor P (phosphoprotein), N binds the viral polymerase (L) to form with the viral RNA the ribonucleoprotein complex (RNP) responsible for transcription and replication of the viral genome. The matrix protein (M) coils and condenses the RNP complex and suppresses transcription of the host genome (Ke et al., 2017). The surface glycoprotein (G) is the main antigenic protein that binds to the cell surface permitting virus attachment and entry (Béarzotti et al., 1995; Lorenzen et al., 1999b). The genome of VHSV also encodes the non-virion protein (NV), which is exclusive to the *Novirhabdovirus* genus and has been shown to be required for *in vivo* pathogenicity (Biacchesi et al., 2010; Ammayappan et al., 2011) due to its role in inhibiting cell apoptosis and modulating the host immune response (Ammayappan and Vakharia, 2011; Kim and Kim, 2013; Biacchesi et al., 2017; Chinchilla and Gomez-Casado, 2017).

The nucleotide sequence encoding the G protein has been widely employed as the preferred molecular target to assess the genetic diversity and evolution of VHSV (Einer-Jensen et al., 2004; Kahns et al., 2012; He et al., 2014; Cieslak et al., 2016; Ghorani et al., 2016; Schönherz et al., 2018), although the N gene has also been adopted (Snow et al., 2004; Einer-Jensen et al., 2005). The phylogenetic studies undertaken so far have identified four genotypes over the Northern Hemisphere (I, II, III, and IV) encompassing nine subtypes (Ia, Ib, Ic, Id, Ie, IVa, IVb, IVc, IVd) and a number of clades (Einer-Jensen et al., 2004; Kahns et al., 2012; Pierce and Stepien, 2012; Vendramin et al., 2019).

In susceptible fish species, VHSV causes a severe systemic disease (i.e., the viral hemorrhagic septicemia, VHS), that often results in high mortality. Until the 1980s, the disease was thought to be restricted only to rainbow trout (*Oncorhynchus mykiss*) reared in freshwater. However, active and passive surveys have revealed that VHSV affects also other marine and freshwater fish, both farmed and wild, raising to more than 110 the number of species with evidence of susceptibility to infection (Skall et al., 2005; Anonymous, 2019; Dadar, 2020). VHSV plasticity in terms of host tropism, is a matter of concern because of the risk of host-jumps from wild fauna to fish species of relevance for the aquaculture industry, and in particular to rainbow trout. Indeed, it was shown that subtypes of VHSV adapted to rainbow trout reared in freshwater share a common ancestor with those subtypes from marine fish species, thus suggesting the occurrence of at least one successful spill over event that resulted in the establishment of subtypes Ia and Ic (Einer-Jensen et al., 2004; He et al., 2014; Schönherz et al., 2018).

In Europe, rainbow trout-adapted subtype Ia is responsible for the majority of disease outbreaks affecting this species, with severe sanitary and economic consequences (Einer-Jensen et al., 2004; Cieslak et al., 2016; Schönherz et al., 2018). In contrast, marine isolates of genotypes II and III, and of subtype Ib showed little or no pathogenicity in rainbow trout under experimental conditions (Skall et al., 2004; Ito et al., 2016). However, subsequent laboratory trials demonstrated that the degree of virulence of isolates belonging to subtype Ib and genotype III in rainbow trout depends, at least to some extent, on the route of infection (Campbell et al., 2009; Dale et al., 2009; Ito et al., 2018). Indeed, the survival rate of intra-peritoneal injected fish was lower, compared to that of bath challenged animals, highlighting that viral entry is critical for pathogenesis. Nevertheless, host adaptation, meaning the capacity of the pathogen to recognize, enter the cells and spread to target organs, is just one of the possible mechanisms that account for virulence. In addition to this, pathways involving genome replication and transcription, assembly and release of virions, and the interaction between viral and host macromolecules (e.g., those involved in the recruitment of cellular factors or in the inhibition of host antiviral defense) can also modulate virulence phenotype and host tropism. Thus, viral proteins and genetic motifs engaged in these processes might be virulence determinants (Baron et al., 1996; Cann, 2012). Consequently, in order to recognize molecular markers modulating VHSV pathogenicity and to understand their synergies, analytical approaches based on the study of the whole genome would be more informative than relying on individual genes.

Two studies carried out using the gain- or loss-of-function approach through reverse genetics, showed that the glycoprotein, the non-virion protein and the polymerase are not virulence determinants for VHSV by themselves, nor in combination (Einer-Jensen et al., 2014; Yusuff et al., 2019), although some authors disagree with this hypothesis (Kim et al., 2014; Baillon et al., 2017). In another study, the phenotype characterization of chimeric viruses resulting from the exchange of the N and P genes of viral clones with different pathogenicity revealed that the N gene plays an essential role in VHSV virulence to rainbow trout. More interestingly, the complete gain of function of chimeric viruses could have been achieved when N was combined with the P gene from the same ancestry (Vakharia et al., 2019). One common denominator of some of the above mentioned studies aiming to identify virulence markers in VHSV genome, is that they are based on the comparison of only two phylogenetically distant isolates (Vakharia et al., 2019; Yusuff et al., 2019). In addition, the virulence of chimeric VHSV was only evaluated *in vivo* by intra-peritoneal injection, which bypasses the natural infection barriers (Campbell et al., 2009; Dale et al., 2009; Ito et al., 2018). A recent study based on sequences comparison of 14 VHSV with different virulence phenotypes in trout has demonstrated that a unique amino acid change in the NV protein, repeatedly observed in several isolates from non-salmonid fish, had been the cause of a strong attenuation of mortality in rainbow trout (Baillon et al., 2017). Thus, it is likely that VHSV possesses different virulence mechanisms in rainbow trout, with other proteins possibly contributing to virulence and to the determination of the clinical outcome.

The combination of phenotype data and complete viral genomes through appropriate statistical models appears to be one of the most effective strategies to understand the molecular basis for VHSV virulence. However, a comprehensive study of this kind, which takes into account the genetic variability of VHSV is still lacking. To bridge this gap, within the Novimark project (Anhiwa ERA-Net) we generated the largest dataset of *in vivo* virulence records and associated whole genome sequences comprising 55 VHSV isolates from most of the known VHSV genotypes and subtypes. We used statistical association models that correlate phenotypic and genetic data to identify amino acid changes (i.e., single amino acid polymorphisms, SAPs) conserved within discrete virulence categories and, as such, potentially involved in VHSV virulence in trout. Secondly, we recognized specific amino acid signatures associated with different virulence phenotypes and mapped them across VHSV phylogenic reconstruction. This integrated approach allowed us to identify VHSV subtypes with potential for higher virulence in rainbow trout. Importantly, we also recognized 38 SAPs within the VHSV genome that are putative virulence determinants in this fish species. These polymorphic sites are candidates for subsequent validation to confirm their role in modulating virulence phenotype *in vivo*.

## Materials and Methods

### Selection of Viral Isolates

Fifty-five VHSV isolates (Table 1) were gathered together to identify putative virulence markers in the coding regions of the viral genome by coupling *in vivo* virulence data with related genetic sequences. Viruses were selected in order to encompass the largest possible genetic variability, according to previous molecular characterization studies based on analyses of partial genome sequences (Einer-Jensen et al., 2004; Abbadi et al., 2016; Baillon et al., 2017). The virulence phenotype (i.e., cumulative percent mortality in bath-challenged rainbow trout) of 35 strains was experimentally determined during the Novimark project, while 20 additional strains (DK-3345, DK-4635, DK-7054, DK-7300, DK-9895174, DK-5p276, DK-5p405, DK-5p26, DK-5e454, DK-5p393, DK-5p508, DK-5p795, DK-5p263, DK-5p11, DK-5p457, DK-5p785, SE-SVA-1033-9C, NO-2007-50-385, JF-JF00Ehil, M.rhabdo) were included in the analysis based on previous knowledge of their level of virulence (Ito et al., 2004, 2018; Skall et al., 2004; Dale et al., 2009). The collection covered a time period of more than 50 years (1962–2015) and comprised genotypes I (Ia–Ie), III and IVa. Most viruses (36/55) were isolated from rainbow trout (*Oncorhynchus mykiss*), while the remaining isolates originated from *Anguilla anguilla* (1/55), *Clupea harengus* (5/55), *Esox lucius* (1/55), *Gadus morhua* (1/55), *Limanda limanda* (2/55), *Merlangius merlangus* (1/55), *Paralichthys olivaceus* (1/55), *Platichthys flesus* (1/55), *Pleuronectes platessa* (2/55), *Salmo trutta* (2/55) and *Sprattus sprattus* (2/55).

**TABLE 1:** List of VHSV isolates tested *in vivo* and subject to whole genome sequencing.

Isolate	Genotype	Origin	Year	Host	Facility tested	%CPM
F1	I	Denmark	1962	*Oncorhynchus mykiss*	DTU	6.6%
He-70	I-undetermined	Denmark	1970	*Oncorhynchus mykiss*	ANSES	40.9%
FR07/71	Ia	France	1971	*Oncorhynchus mykiss*	ANSES	76.3%
23/75	Ia	France	1975	*Salmo trutta*	ANSES	98.1%
FR02/84	Ia	France	1984	*Oncorhynchus mykiss*	ANSES	89%
1458	Ia	France	1990	*Oncorhynchus mykiss*	ANSES	99.4%
3771P	Ia	France	1990	*Oncorhynchus mykiss*	ANSES	100%
N11298	Ia	France	2003	*Esox lucius*	ANSES	100%
MM73	Ia	France	2014	*Oncorhynchus mykiss*	ANSES	100%
1236-01	Ia	France	2014	*Oncorhynchus mykiss*	ANSES	100%
DK-6137	Ia	Denmark	1991	*Oncorhynchus mykiss*	ANSES	98.1%
DK-3345^a^	Ia	Denmark	1985	*Oncorhynchus mykiss*	DTU	46.1%
DK-3592B	Ia	Denmark	1989	*Oncorhynchus mykiss*	DTU	92%
DK-6435^a^	Ia	Denmark	1992	*Oncorhynchus mykiss*	DTU	71.6%
DK-7054^a^	Ia	Denmark	1993	*Oncorhynchus mykiss*	DTU	56%
DK-7300^a^	Ia	Denmark	1994	*Oncorhynchus mykiss*	DTU	67.5%
DK-9895174^a^	Ia	Denmark	1998	*Oncorhynchus mykiss*	DTU	73.9%
DK-203490	Ia	Denmark	2003	*Oncorhynchus mykiss*	DTU	99.3%
VHSV/O.mykiss/I/TN/480/Oct96	Ia	Italy	1996	*Oncorhynchus mykiss*	IZSVe	14.5%
VHSV/O.mykiss/I/PN/234/Mar99	Ia	Italy	1999	*Oncorhynchus mykiss*	IZSVe	100%
VHSV/O.mykiss/I/BZ/301/Jun00	Ia	Italy	2000	*Oncorhynchus mykiss*	IZSVe	89%
VHSV/O.mykiss/I/TV/3/Dec02	Ia	Italy	2002	*Oncorhynchus mykiss*	IZSVe	55.7%
VHSV/O.mykiss/I/TV/299/Aug04	Ia	Italy	2004	*Oncorhynchus mykiss*	IZSVe	100%
VHSV/O.mykiss/I/TN/475/Nov04	Ia	Italy	2004	*Oncorhynchus mykiss*	IZSVe	94.1%
VHSV/S.trutta/I/TN/470/Nov09	Ia	Italy	2009	*Salmo trutta*	IZSVe	55.7%
VHSV/O.mykiss/I/TN/80/Mar10	Ia	Italy	2010	*Oncorhynchus mykiss*	IZSVe	100%
VHSV/O.mykiss/I/TN/28/Feb11	Ia	Italy	2011	*Oncorhynchus mykiss*	IZSVe	100%
VHSV/O.mykiss/I/TN/62/Feb15	Ia	Italy	2015	*Oncorhynchus mykiss*	IZSVe	100%
VHSV/O.mykiss/I/TN/68/Feb15	Ia	Italy	2015	*Oncorhynchus mykiss*	IZSVe	100%
VHSV/O.mykiss/I/TN/84/Feb15	Ia	Italy	2015	*Oncorhynchus mykiss*	IZSVe	90%
DK-1p8	Ib	Baltic Sea	1996	*Clupea harengus*	ANSES	0%
M.rhabdo	Ib	Baltic Sea	1979	*Gadus morhua*	DTU	0%
DK-5p276^a^	Ib	Kattegat	1998	*Pleuronectes platessa*	DTU	5.2%
DK-5p405^a^	Ib	Baltic Sea	1998	*Limanda limanda*	DTU	8.2%
DK-5p26^a^	Ib	Kattegat	1998	*Limanda limanda*	DTU	8.2%
DK-5e454^a^	Ib	Baltic Sea	1998	*Platichthys flesus*	DTU	5.2%
DK-5p393^a^	Ib	Baltic Sea	1998	*Clupea harengus*	DTU	11.4%
DK-5p508^a^	Ib	Baltic Sea	1998	*Clupea harengus*	DTU	9.7%
DK-5p795^a^	Ib	Baltic Sea	1998	*Clupea harengus*	DTU	5.6%
DK-5p263^a^	Ib	Kattegat	1998	*Clupea harengus*	DTU	5.6%
DK-5p11^a^	Ib	Skagerrak	1998	*Pleuronectes platessa*	DTU	5.2%
DK-5p457^a^	Ib	Baltic Sea	1998	*Sprattus sprattus*	DTU	12.95%
DK-5p785^a^	Ib	Baltic Sea	1998	*Sprattus sprattus*	DTU	4.7%
SE-SVA-1033-9C^b^	Ib	Sweden	2000	*Oncorhynchus mykiss*	DTU	4%
DK-2149	Ic	Denmark	1978	*Oncorhynchus mykiss*	DTU	66%
DK-3612	Ic	Denmark	1986	*Oncorhynchus mykiss*	DTU	18.6%
NO-A163-68-EG46	Id	Norway	1968	*Oncorhynchus mykiss*	DTU	4%
FiP02b.00	Id	Finland	2000	*Oncorhynchus mykiss*	DTU	16%
GE 1.2	Ie	Georgia	1981	*Oncorhynchus mykiss*	DTU	53.3%
Trabzon 207111	Ie	Turkey	2004	*Oncorhynchus mykiss*	DTU	30.6%
2009-50-315-1	Ie	Turkey	2009	*Oncorhynchus mykiss*	DTU	78.6%
FR-L59x	III	France	1987	*Anguilla anguilla*	ANSES	0%
DK-4p101	III	North Sea	1997	*Merlangius merlangus*	ANSES	0%
NO-2007-50-385^c^	III	Norway	2007	*Oncorhynchus mykiss*	DTU	69.1%
JF-JF00Ehil^d^	IVa	Japan	2000	*Paralichthys olivaceus*	DTU	0.9%

*For all the viruses, information on the geographic origin, host and year of isolation, genotype and cumulative percent mortality in experimentally challenged rainbow trout (Oncorhynchus mykiss) are provided. References for viruses tested in vivo in previous studies are reported. ^a^Skall et al. (2004); ^b^Ito et al. (2018); ^c^Dale et al. (2009); ^d^Ito et al. (2004).*

### Viral Batches Production

#### Laboratory 1 – ANSES

Isolates He-70, FR07/71, 23/75, FR02/84, 1458, 3771P, N11298, MM73 and 1236-01 were propagated in bluegill fry (BF-2) cell monolayers (ATCC^®^ CCL91TM) according to standard procedures (Lorenzen et al., 1999a; Anonymous, 2019). In detail, 1 ml of each viral strain was added to 75 cm^2^ polystyrene flasks (Falcon^®^) seeded with 24-h BF-2 cells and adsorbed for 1 h at 14 ± 1°C. After adsorption, 12 ml of pH 7.6 Tris-buffered L15 medium (Gibco) supplemented with 10% fetal calf serum (FCS, BioWest), 1% L-glutamine 200 mM (HycloneTM) and 1% penicillin–streptomycin solution 100X (PanTM Biotech) were added to each flask and viruses were incubated at 14 ± 1°C until completion of cytopathic effect (CPE). For each strain, cell culture supernatant was then collected, clarified at 4°C for 15 min at 2000 × *g* and checked by IFAT (Afnor, 2010; Anonymous, 2019). Viral stocks were titrated in 96-well plates using the 50% tissue culture infectious dose (TCID_50_) endpoint method (Kärber, 1931). Viral batches were finally aliquoted and stored at −80°C until use.

#### Laboratory 2 – DTU

Isolates F1, NO-A163-68-EG46, DK-2149, M.rhabdo, GE 1.2, DK-3612, DK-3592B, FiP02b.00, DK-203490, Trabzon 207111, and 2009-50-315-1 from the VHSV repository of the EURL for Fish and Crustacean Diseases were propagated in BF-2 cell monolayers in 25 cm^2^ flasks at 15°C for 7 days. Supernatant from the flasks was harvested, filtered using 0.45 μM Minisart^®^ syringe filters (Sartorius) to remove cell debris and stored at −80°C until use.

Viruses were subject to plaque purification using methylcellulose in order to produce a homogeneous viral population. Briefly, all propagated isolates were ten-fold serially diluted and incubated with BF-2 cells monolayers in 96-well trays for 1 h at 15°C. The viral inoculum was removed and a mixture of methylcellulose and cell culture medium (Eagle’s MEM with L-glutamine, penicillin–streptomycin, Tris-HCl and 2% bovine fetal serum, Gibco) was added to each well. Cells were incubated at 15°C for 7 days and monitored for plaques formation. Due to the liquid nature of methylcellulose, plaques were collected only from wells containing single plaques to avoid mixed viral populations. Collected plaques were propagated in 75 cm^2^ flasks containing BF-2 monolayers, harvested as described above and frozen at −80°C in aliquots for subsequent titration, full genome sequencing and use in infection trials (see below). Determination of viral titer of the isolates was calculated through the method of Spearman-Karber (Kärber, 1931). Information regarding processing of the remaining isolates is available in the respective papers (Ito et al., 2004, 2018; Skall et al., 2004; Dale et al., 2009).

#### Laboratory 3 – IZSVe

All Italian VHSV isolates were ten-fold serially diluted (from 10^–1^ to 10^–6^) with MEM Eagle (Sigma-Aldrich) containing 2% FCS (Hyclone), 2 mM L-glutamine (Sigma-Aldrich) and 1X antibiotic/antimycotic (Sigma-Aldrich). Six-hundred microliters of each dilution were inoculated into Falcon^®^ 12-well polystyrene culture plates (Corning) seeded with confluent 24-h-old EPC cells. Viral inoculum was adsorbed at 15°C for 3 h under gentle shaking and then removed. Subsequently, 2 ml of a 1:3 solution of 2% FCS-MEM Eagle (Sigma-Aldrich) and carboxymethyl cellulose (Sigma-Aldrich) were added to each well. Plates were then incubated at 15°C and checked daily for cytopathic effect. Upon observation of CPE, for each strain 10 μl of supernatant were collected from discrete plaques with defined edges with the aid of a pipette under the light microscope. Viruses originating from individual plaques were subject to two additional plaque-purification rounds as described above. Each viral clone was then propagated in 75 cm^2^ polystyrene flasks (Falcon^®^) seeded with 24-h-old EPC cells. Briefly, viruses were adsorbed for 1 h at 15°C under gently shaking. After adsorption, 15 ml of 10% FCS-MEM Eagle (Sigma-Aldrich), 2 mM L-glutamine (Sigma-Aldrich) and 1X antibiotic/antimycotic (Sigma-Aldrich) were added to each flask and viruses were incubated at 15°C until complete CPE. For each strain, cell culture supernatants were collected, clarified for 10 min at 2800 × *g* at 4°C, and checked by real-time reverse-transcription polymerase chain reaction PCR (rRT-PCR) (Jonstrup et al., 2013) and by G-gene sequencing (Abbadi et al., 2016). Viral titer was expressed as TCID_50_/ml according to the Reed and Müench formula (Reed and Müench, 1938). Viral batches were divided into aliquots and stocked at −80°C until use.

### Experimental Challenges

#### Laboratory 1 – ANSES

The experimental protocol for fish challenges was conceived in compliance with the Directive 2010/63/EU and the transposition texts published in the Official Journal of the French Republic on the 6th January 2012 and on the 7th February 2013. The experimental design was evaluated by ANSES Ethics Committee (ANSES/ENVA/UPC n° 16) and finally approved by the Ministère de l’Éducation Nationale, de l’Enseignement Supérieur et de la Recherche, with the authorizations n° 08/04/14-10 and 14/06/16-8 (APAFiS: 2016053117453469).

*In vivo* tests were performed using pathogen-free rainbow trout of approximately 3 g originating from ANSES breeding facilities. Fish were maintained in filtered freshwater at a temperature of 10 ± 2°C and fed once a day with commercial feed. Infection trials were conducted in triplicate using 10 L tanks containing 50 fish each. Fish were exposed for 3 h to a static water 4 L bath containing 10^4^ TCID_50_/ml of virus under enhanced aeration. One additional group (three tanks) was mock-infected with sterile L15 medium (Gibco) applying the same conditions. At the end of the challenge, water level was restored up to 10 L by adding clean freshwater and the flow was turned on (open water system, 10 L/h). Fish were monitored regularly for 32 days, and daily mortality was recorded. At the end of the observation period, survivor fish were euthanized by anesthetic overdose (Eugenol, Fili@vet). Bacteriological and virological analyses were conducted on dead fish as confirmatory diagnosis.

#### Laboratory 2 – DTU

Experimental infections were performed in accordance with the current animal welfare regulations (Directive 2010/63/EU) and approved by the Danish Animal Research Authority under the license 2013-15-2934-00976.

Rainbow trout (average size 1 g) reared at DTU facilities were transferred into aerated freshwater 8 L capacity tanks with constant flow-through system and 300% water renewal per day. Housing conditions were: 12 ± 1°C, a light to dark ratio of 12:12, a stocking density ≤ 70 kg/m^3^, feeding 1.5% of biomass. For each strain (i.e., F1, NO-A163-68-EG46, DK-2149, GE 1.2, DK-3612, DK-3592B, FiP02b.00, DK-203490, Trabzon 207111, 2009-50-315-1) the experimental infection was done in triplicate tanks containing 50 fish each. Challenge groups were bath-exposed for 5 h to 10^5^ TCID_50_/ml of each VHSV isolate, except for the isolate DK-203490, whose infection dose was 10^4^ TCID_50_/ml. Three additional 50-fish tanks were used to mock-infect animals with Eagle’s Essential Media (Gibco) with Tris-HCl and 10% fetal calf serum (Gibco), under the same conditions as above. The trial was terminated after 30 days. Fish were monitored daily for mortality, as well as for their welfare status. Animals presenting clinical signs (i.e., apathy, skin darkening, exophthalmos and swimming abnormal behavior) were euthanized by immersion in benzocaine chloride (800 mg/L) (Sigma-Aldrich), pooled by day and tank, and tested for VHSV by re-isolation in cell culture and ELISA identification (Anonymous, 2019).

*In vivo* virulence of strain M.rhabdo was assessed at the facilities of DTU in Aarhus (Denmark) in December 1990 and is presented here for the first time. Rainbow trout of 3 g were housed in duplicate 8 L tanks containing 40 fish each and exposed by immersion for 2 h to 10^5^ TCID_50_/ml of virus. Two additional tanks were used to mock-challenge negative control fish with MEM (Minimum Essential Medium) containing 10% fetal bovine serum. Daily mortality was recorded for a 36-day period.

Additional *in vivo* virulence data related to 19 strains (i.e., DK-3345, DK-4635, DK-7054, DK-7300, DK-9895174, DK-5p276, DK-5p405, DK-5p26, DK-5e454, DK-5p393, DK-5p508, DK-5p795, DK-5p263, DK-5p11, DK-5p457, DK-5p785, NO-2007-50-385, SE-SVA-1033-9C, JF-JF00Ehil) were retrieved from previous papers (Skall et al., 2004; Ito et al., 2004, 2018; Dale et al., 2009) and used as metadata.

#### Laboratory 3 – IZSVe

The experimental protocol for fish challenges was designed in compliance with the Directive 2010/63/EU and the national Legislative Decree No. 26/2014. The experimental design was evaluated by the IZSVe Animal Welfare Body and Ethics Committee and finally approved by the Italian Ministry of Health with the authorization n° 735/2016-PR 22/7/2016.

The sample size for experimental challenges was determined by conditional Fisher’s exact test for two proportions with Walters’ normal approximation, assuming an α error of 0.05 (one tail) and power 1-β of 0.90 (SAS software).

Rainbow trout juveniles of approximately 0.6 g were purchased from an Italian commercial farm classified within Category I (EU Directive 88/2006). Fish were housed for 10 days in a 2500 L tank at 10 ± 2°C for acclimation, and fed with commercial feed. After this period, 12 groups of 70 specimens each were moved into individual tanks and challenged by immersion. Exposure with 10^4^ TCID_50_ of each viral strain per ml of water was performed for 3 h in 20 L static water supplying additional aeration. One extra group was mock-infected with sterile MEM Eagle (Sigma-Aldrich) under the same conditions. At the end of the challenge, water level was resumed to 80 L by adding clean freshwater and the flow was turned on (open water system, 10 L/h). Fish were monitored regularly for a 4-week period, and daily mortality was recorded. At the end of the observation period, survivor fish were euthanized by anesthetic overdose (Tricaine, Pharmaq). Confirmatory diagnosis for VHSV detection was performed on dead specimens by rRT-PCR (Jonstrup et al., 2013).

### Whole Genome Sequencing

All the viruses were subjected to high-throughput sequencing (HTS) to obtain their complete genome sequences. The isolates were sequenced by three different laboratories (ANSES, CEFAS, IZSVe) using two technologies, i.e., Illumina MiSeq (*n* = 28) and IonProton^TM^ (*n* = 27). Sample processing procedures, as well as sequencing and bioinformatic (BI) analyses are reported in detail in Zamperin et al. (2019). To assure data comparability among different laboratories performing HTS, a proficiency test focusing on the BI pipelines was carried out as described in the same paper. The test proved that the BI analyses of the three laboratories were 99.98% accurate and 99.94% repeatable.

Genome annotation was performed using a Blast-like algorithm^1^ (version 11.1.5) to identify regions with a similarity >80% to isolate 96-43 (Betts and Stone, 2000) available under the GenBank accession number AF143862.1. Coding regions were translated to protein sequences and visually inspected for shifts in the open reading frames (ORFs) and to detect premature/delayed stop codons.

For each strain, we produced a nucleotide (nt) sequence encompassing the complete open reading frame (ORFs) of every gene, without stop codons, later referred to as “concatamer.” The nt-concatamers, and their deduced amino acid (aa) sequences (aa-concatamers) were used for downstream analyses.

### Phylogenetic Analysis

Full genome sequences were aligned using MAFFT v7.388 (Katoh, 2002; Katoh and Standley, 2013), with default settings. Two different datasets were created, the first including the whole genome sequences of VHSV isolates, except for the 3′ and 5′ UTRs (10,863 characters). In the second matrix, the nt-concatamers were included (10,338 characters). Phylogenetic analyses were carried out in both matrixes using RAxML v.8 (Stamatakis, 2014) with the GTR+G model, and all free parameters estimated by the program, with 1000 rapid bootstrap inferences and a search for the best-scoring maximum likelihood (ML) tree.

An additional phylogenetic reconstruction based on the nt-concatamer was carried out with IQ-Tree (Nguyen et al., 2015; Trifinopoulos et al., 2016) using the best-fitting nucleotide substitution model as estimated by ModelFinder (Kalyaanamoorthy et al., 2017), implementing the Bayesian Information Criterion (BIC), and with 1,000 replicates of the ultrafast bootstrap approximation (Hoang et al., 2018).

### Statistical Analyses

All the analyses herein described were carried out using the R statistical programming environment (R Development Core Team, 2018).

#### Survivorship Analyses

Mortality data were analyzed by plotting Kaplan–Meier survival curves for all the isolates tested *in vivo* (*n* = 35) using the functions implemented in the package *survival* (Therneau, 2015).

#### Identification of VHSV Virulence Classes

Based on the multi-sequence alignment of the aa-concatamers, SAPs with a minimum frequency of 3% were selected. Among these SAPs, those significantly associated with variations in the cumulative percent of mortality (CPM) were identified applying a generalized linear model. The model assumed a quasi-binomial distribution of the mortality values to account for their over-dispersion. The strength of the association was then determined via a likelihood ratio test. SAPs for which we observed a *p* < 10^–4^ (significance limit = alpha/SAPs number = 0.05/499 ≈ 10^–4^) were considered statistically significant. Virulence classes were then defined based on CPM thresholds that maximized the genetic homogeneity among VHSV strains. In detail, for each polymorphism associated to CPM we conducted an iterative analysis in which at each cycle: (i) a CPM threshold (range: 1–99%; stepwise increase: 1%) identifying two viruses partitions was established; (ii) a contingency table reporting the number of strains segregating into each partition and displaying any possible aa signature was generated; (iii) Fisher’s exact test was performed on the contingency table to assess the dependency between the aa signatures and the two identified partitions; (iv) the observed *p*-value was acquired if smaller than the expected significance limit (alpha/number of tested thresholds = 0.05/99 ≈ 5 × 10^–4^). For each SAP, the CPM value associated with the smallest *p*-value was selected. The frequency of all candidate thresholds identified as above were plotted as histograms, and those showing the highest number of observations were used to designate three virulence classes, i.e., high “H”, moderate “M” and low “L”. Amino acid sequence homogeneity among VHSV strains belonging to the same virulence class were assessed by a quasi-binomial generalized linear model relating CPM to the variants observed at each SAP. Moderate viruses were not tested for their homogeneity because of dataset size constraints. The strength of the association was determined via a likelihood ratio test and the resulting *p*-value was retained only if smaller than 1.3 × 10^–4^ in the case of “H” (number of SAPs: 367) or 1.8 × 10^–4^ in the case of “L” (number of SAPs: 274).

#### Discovery of SAPs Implicated in Virulence and Host Tropism

Analyses were conducted in the context of conditional inference based on permutation tests with the package *coin* (Hothorn et al., 2008). In detail, the association between SAPs and the trait “virulence” (categorized as high/moderate/low) was evaluated by Cochran-Armitage or extended Cochran-Armitage test, for polymorphic sites presenting two or more than two different signatures in the aa-concatamers alignment, respectively. The association between SAPs and the variable “host tropism” (categorized as rainbow trout/other) was evaluated by Pearson’s chi square test. In all cases, *p* < 10^–4^ was considered significant (see above).

The correlation between “virulence” and “host tropism” was assessed with the Cramer’s index V, determined with the package *vcd* (Meyer et al., 2017). In order to infer the strength of the association of the variables “virulence” and “host tropism” to the SAPs herein identified, and to determine whether such association was significant for both traits or if a significant association between traits was present, a binomial or multinomial logistic regression model was constructed for each polymorphic site to relate the frequency of each amino acidic signature to both the aforementioned variables. In case of complete or quasi-complete separation, the regression model was based on penalized maximum likelihood implemented by the package *brglm2* (Kosmidis, 2019). For both variables, the strength of the association was then determined via a likelihood ratio test.

#### Identification of Amino Acid Signatures Associated to Different Virulence Phenotypes

For the SAPs being relevant for virulence, we estimated the frequency of all the aa signatures within the different virulence classes previously identified. Based on these estimates, a pairwise proportion test was conducted to determine which of the aa signatures were significantly associated with the high, moderate and low virulence classes (package *EnvStats*) (Millard, 2013). The observed *p*-values were adjusted using the Bonferroni’s method and considered statistically significant when smaller than 0.05.

## Results

### Whole Genome Sequencing

In this study, we successfully sequenced the entire genome of 55 VHSV isolates. The average coverage ranged between 1,015 and 32,372X and was sufficient for variant calling and for producing high quality consensus sequences. Notably, the low coverage at the 3′ and 5′ ends of the viral genome (leader and trailer, respectively) impeded the assignment of unambiguous nucleotides (nt) in these regions and their full reconstruction, as previously reported (Zamperin et al., 2019). Illumina MiSeq and Ion Proton^TM^ raw data are available through NCBI’s Sequence Read Archive (SRA^2^). Consensus sequences can be retrieved from GenBank^3^ and the Fishpathogens database^4^ (Supplementary Table S1).

Annotation revealed the typical VHSV genome architecture, with six genes coding the N, P, M, G, NV and L proteins. Relatively numerous genome size differences among isolates were found at the intergenic regions because of the presence of deletions with variable extension. In contrast, only two peculiarities were found in the coding regions of the dataset analyzed in this study, both harbored by isolate VHSV/S.trutta/I/TN/470/Nov09. In the genome of this isolate, a premature stop codon was found in the M gene, rendering its deduced amino acid (aa) sequence eight residues shorter than expected. Additionally, if compared with the other isolates included within this study, the NV aa sequence of this isolate showed an extra eight residues near the C-terminus. Whether the size differences in both proteins had any influence on the phenotype of VHSV/S.trutta/I/TN/470/Nov09 was not further investigated.

Notably, as the presence of deletions within the intergenic regions impeded the comparison of all the isolates at these portions of the genome, their use in downstream association analyses was avoided. Thus, the search for VHSV virulence markers described below was carried out exclusively within the coding regions (i.e., aa-concatamers).

### VHSV *in vivo* Phenotypes and Identification of Virulence Classes

**FIGURE 1:**
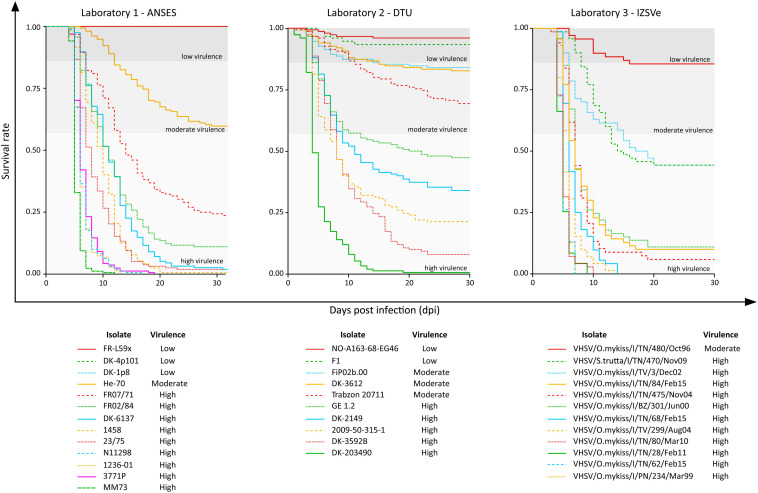
Kaplan–Meier curves of the 35 VHSV isolates tested for their virulence in rainbow trout in the current study. The *y*-axis reports the survival rate, and the *x*-axis reports the observation period expressed as days post infection (dpi). In each graph, the thresholds that distinguish the high “H” (CPM > 42%), moderate “M” (14% < CPM ≤ 42%) and low “L” (CPM ≤ 14%) virulent classes as assessed in this study are also reported.

Survival rate curves were plotted for the 35 VHSV isolates tested *in vivo* during the Novimark project (Figure 1). For the remaining strains, we referred to the specific publications (Ito et al., 2004, 2018; Skall et al., 2004; Dale et al., 2009). Altogether, these data pointed out a variety of different virulence phenotypes induced by this panel of isolates in experimentally challenged rainbow trout. Apart from the avirulent strains FR-L59x, DK-4p101 and DK-1p8, the first mortality records could be observed between 1 and 6 days post infection (dpi), depending on the isolate. Upon visual inspection of the Kaplan–Meier curves, it appeared that the viruses were clustered in different groups, based on survival rates over time. In general, the most lethal viruses determined a sharp decrease in fish survival during the first 10–15 days after challenge. On the contrary, less virulent viruses showed curves with a different trend, characterized by a slow and low decrease of the survival rate over time. Other viruses showed an intermediate behavior between these two groups, with no clear pattern.

**FIGURE 2:**
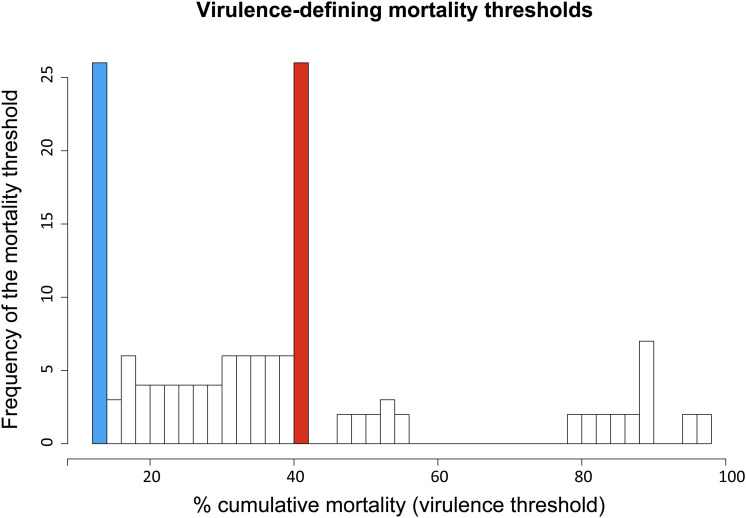
Frequency distribution of virulence-defining mortality thresholds. Each value derived from an iterative analysis conducted on SAPs associated to cumulative mortality variation. Ninety-nine different mortality thresholds, within the range 1–99%, were tested to identify the value maximizing VHSV strains segregation in terms of cumulative percent of mortality (CPM) and aa-signature. The CPM thresholds defining the virulence classes “L” (CPM ≤ 14%) and “H” (CPM > 42%) are highlighted in blue and red, respectively.

Overall, the cumulative percentage of mortality recorded within the whole dataset (55 strains) was highly variable, ranging between 0% and 100% (Table 1). To assess the phenotypic differences observed in our virus collection using a more rigorous methodology, and to categorize each strain in terms of virulence, we used CPM and available protein sequence data. In detail, when considering the variable sites in the aa-concatamer multi-sequence alignment, we identified 41 SAPs that changed significantly among strains in response to variation in mortality (*p* < 10^–4^) (Supplementary Table S2). For each of these SAPs, we determined the CPM value that maximized polymorphism segregation. To do so, we used a simulation process that allowed viral partitioning into two distinct groups based on CPM cut offs. The mortality thresholds observed with the highest frequency among all 41 SAPs were adopted to outline virulence classes (Figure 2). Our data showed that all the viruses associated with a CPM of more than 42% (31 out of 55) were homogeneous at their aa sequences, when compared with isolates which caused a lower mortality (Supplementary Figure S1). This cluster of viruses was labeled as high virulent “H”. Similarly, isolates associated with a CPM of less or equal to 14% (19 out of 55) were characterized as having high aa homogeneity (Supplementary Figure S2) and were designated as low virulent strains “L”. Five isolates that did not fall into any of the “H” or “L” groups showed CPM > 14% and ≤42%, and were arbitrarily defined as moderate virulent strains “M”.

### Identification of SAPs Associated to Virulence and Host Tropism

**FIGURE 3:**
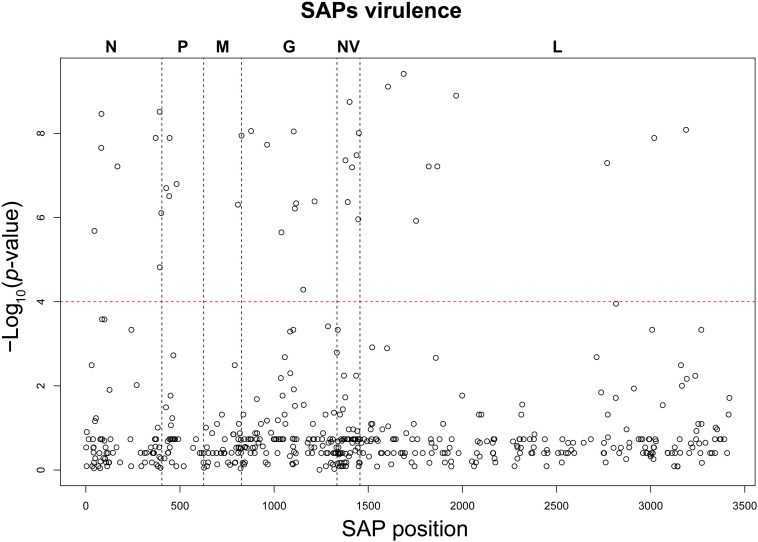
Manhattan plot of the association analysis conducted on VHSV SAPs and the trait “virulence.” The SAP position along the genome coding regions is displayed on the *x*-axis, while the *y*-axis reports the negative logarithm of the association *p*-value. The red dashed line identifies the association test significance limit.

Based on the establishment of the “H”, “M”, and “L” virulence classes, we recognized 38 SAPs among the variable sites in the aa multi-sequence alignment that varied significantly among the three virulence groups (*p* < 10^–4^) (Table 2). These SAPs, putatively linked to VHSV virulence in trout and later referred to as “SAPs-virulence,” were scattered throughout the entire coding regions of VHSV (Figure 3). Eight SAPs-virulence were in the N protein, 4 in P, 2 in M, 8 in G, 7 in NV, and 9 in L. If taking into account the relative length of each protein, it was noticeable that the majority of SAPs-virulence were located in the nucleoprotein, the glycoprotein and the non-virion protein sequence.

**TABLE 2:** Single amino acid polymorphisms (SAPs) associated to virulence and host tropism.

		Frequency of aa signatures (%) associated to				
		Virulence			Host tropism	
SAP	Variants	Low	Moderate	High	Rainbow trout	Other
N46	E	0	20	0	2.9	0
	G	89.5	40	12.9	17.1	85
	K	0	0	16.1	8.6	10
	R	10.5	40	71	71.4	5
N82	E	21.1	100	96.8	91.4	35
	G	78.9	0	3.2	8.6	65
N83	A	15.8	0	3.2	2.9	15
	M	73.7	0	0	2.9	65
	T	10.5	100	96.8	94.3	20
N168	H	31.6	100	100	97.1	40
	Y	68.4	0	0	2.9	60
N371	K	26.3	100	100	97.1	35
	R	73.7	0	0	2.9	65
N392	E	94.7	60	9.7	20	85
	G	5.3	40	90.3	80	15
N393	E	5.3	40	67.7	-	-
	G	94.7	60	32.3	-	-
N401	E	26.3	80	93.5	91.4	30
	G	73.7	20	6.5	8.6	70
P23	K	94.7	60	19.4	-	-
	R	5.3	40	80.6	-	-
P39	A	0	20	3.2	2.9	5
	N	0	0	6.5	5.7	0
	P	73.7	0	0	2.9	65
	S	5.3	0	0	0	5
	T	21.1	80	90.3	88.6	25
P41	E	73.7	0	0	2.9	65
	G	26.3	100	100	97.1	35
P78	F	21.1	80	93.5	88.6	30
	L	78.9	20	6.5	11.4	70
M182	I	10.5	60	51.6	-	-
	L	0	0	6.5	-	-
	M	15.8	20	41.9	-	-
	T	73.7	20	0	-	-
M201	Q	5.3	20	3.3	2.9	10.5
	R	94.7	40	10	22.9.	79
	W	0	40	86.7	74.3	10.5
G51	D	0	20	87.1	-	-
	E	94.7	80	12.9	-	-
	K	5.3	0	0	-	-
G136	D	0	20	80.6	68.6	10
	N	100	80	19.4	31.4	90
G212	E	0	0	16.1	-	-
	K	0	20	61.3	-	-
	N	0	0	3.2	-	-
	Q	5.3	20	0	-	-
	T	94.7	60	19.4	-	-
G277	A	89.5	80	9.7	-	-
	E	10.5	0	3.2	-	-
	T	0	20	87.1	-	-
G283	K	100	60	22.6	-	-
	N	0	20	67.7	-	-
	R	0	20	9.7	-	-
G290	I	94.7	80	22.6	-	-
	V	5.3	20	77.4	-	-
G328	I	100	80	45.2	-	-
	V	0	20	54.8	-	-
G388	D	26.3	80	87.1	85.7	30
	G	5.3	20	12.9	11.4	10
	N	68.4	0	0	2.9	60
G506	M	-	-	-	34.3	95
	T	-	-	-	65.7	5
NV45	M	68.4	60	9.7	-	-
	R	26.3	20	3.2	-	-
	T	5.3	0	0	-	-
	V	0	20	87.1	-	-
NV57	D	26.3	100	87.1	88.6	30
	E	5.3	0	12.9	8.6	10
	N	68.4	0	0	2.9	60
NV67	H	0	40	87.1	74.3	15
	Y	100	60	12.9	25.7	85
NV80	G	5.3	40	48.4	45.7	10
	K	73.7	0	0	2.9	65
	R	21.1	60	51.6	51.4	25
NV104	F	5.3	0	0	-	-
	I	94.7	80	16.1	-	-
	V	0	20	83.9	-	-
NV113	I	78.9	80	9.7	-	-
	L	5.3	20	83.9	-	-
	P	0	0	3.2	-	-
	T	5.3	0	0	-	-
	V	10.5	0	3.2	-	-
NV116	N	15.8	0	3.2	-	-
	R	0	20	87.1	-	-
	S	84.2	80	9.7	-	-
L149	E	10.5	60	96.8	88.6	20
	G	89.5	40	3.2	11.4	80
L232	I	0	40	90.3	-	-
	V	100	60	9.7	-	-
L298	E	89.5	80	16.1	-	-
	K	10.5	0	83.9	-	-
	R	0	20	0	-	-
L365	I	31.6	100	100	97.1	40
	V	68.4	0	0	2.9	60
L411	F	31.6	100	100	97.1	40
	Y	68.4	0	0	2.9	60
L511	K	100	80	12.9	28.6	85
	R	0	20	87.1	71.4	15
L1313	A	0	20	3.2	-	-
	L	0	0	3.2	-	-
	M	0	20	67.7	-	-
	T	100	60	9.7	-	-
	V	0	0	16.1	-	-
L1563	I	73.7	0	0	2.9	65
	L	26.3	100	100	97.1	35
L1732	A	0	20	87.1	-	-
	K	0	20	0	-	-
	T	100	60	12.9	-	-

*For each SAP, amino acid variants and their frequencies expressed as percentage are reported for all the categories in our dataset of traits “virulence” (low; moderate; high) and host tropism (rainbow trout; other). A hyphen (-) indicates that a particular SAP is neither associated with virulence nor with host tropism.*

To understand whether VHSV virulence might be related to host tropism, we applied the same analytical criteria as above to identify polymorphic sites (later referred as “SAPs-host”) that showed significant variations among isolates in respect to the host of origin, classing such isolates as either originating from rainbow trout or from other species (Table 2 and Supplementary Figure S3). Twenty-two SAPs-host putatively linked to the host of origin were subsequently identified. Notably, all of them were associated also with virulence, with the exception of G506, which was unique to this category. To rule out any possible bias in our analysis, we assessed the level of collinearity of the variables virulence and host tropism. The Cramer index V revealed that virulence and host tropism were, at least to some extent, co-dependent (0.73). However, a multi-trait association analysis using logistic binomial/multinomial models revealed the existence of SAPs (N46, N401, L149) significantly associated with both variables, individually, indicating that virulence and host tropism were sufficiently independent from each other to be considered simultaneously in the same model. Thus, we suggest that such polymorphisms are VHSV virulence markers acting as determinants of host tropism. In addition, the same analysis showed that, among SAPs that initially appeared associated with both traits (*n* = 21), all but N46, N401, and L149 could be considered significantly associated only to the trait “virulence” (Supplementary Table S3).

### Identification of Amino Acid Signatures Associated to Virulence Phenotypes

For all the 38 SAPs-virulence, we estimated the association of the aa signatures observed at the polymorphic sites with the virulence classes “H”, “M”, and “L”. Significant associations (*p* < 0.05) were observed only in relation to the high and low virulent groups, while no aa signature was characteristic of moderate group (Supplementary Table S4).

**FIGURE 4:**
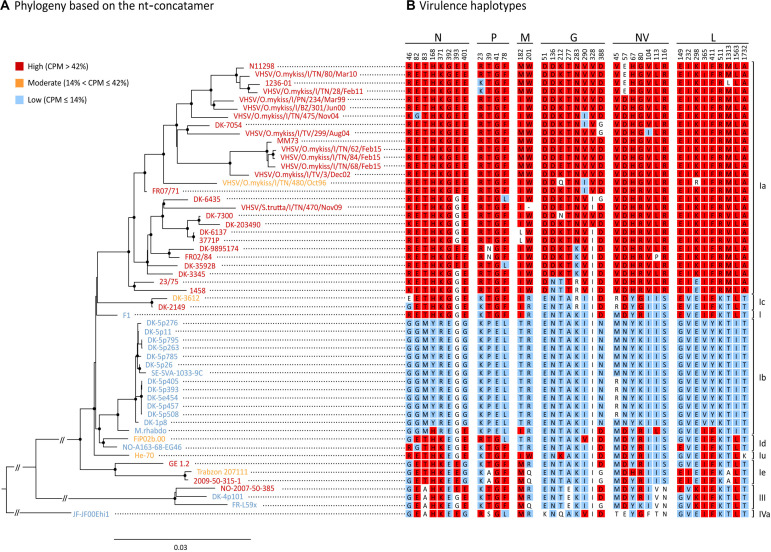
Phylogenetic reconstruction of the 55 isolates used in this study. **(A)** ML phylogenetic tree based on the nt-concatamer set. Isolates are color-coded depending which virulence class they belong to, where red is high (“H”, CPM > 42%), orange is moderate (“M”, 14% < CPM ≤ 42%) and blue is low (“L”, CPM ≤ 14%). Nodes with bootstrap support ≥ 70% are labeled with a dot. Branch lengths are scaled according to the number of nucleotide substitutions per site. Vertical bars on the right indicate genotypes/subtypes subdivision. **(B)** Amino acid residues at SAPs-virulence (i.e., virulence haplotypes) are shown for each isolate, indicating signatures associated either to the high (in red), or to the low virulence class (in blue) (*p* < 0.05). Amino acids with no significant association to any of the virulence classes are showed in white.

Based on this association analysis, it was possible to identify, for all the VHSV herein studied, the presence of amino acid residues associated with the “H” or “L” phenotypes at the 38 SAPs implicated in virulence. The combination of amino acids obtained for all the strains is hereafter referred to as the “virulence haplotype.” Overall, we observed 23 unique virulence haplotypes out of 31 “H” strains, 5 “M” haplotypes among the five isolates of this group, and nine unique haplotypes within the 19 “L” viruses (Figure 4).

### Phylogenetic Reconstruction

Overall, the level of similarity observed among the 55 isolates based on the nt-concatamer ranged between 86 and 100%. The percentage of similarity of individual clusters ranged between 95.7 and 100% for Ia, 99.2–100% for Ib, 97.5–99.8% for Ie, and 98.1–98.5% for genotype III, as well as being measured at around 99.7% and 99.2% for types Ic and Id, respectively, neither of which was represented by more than two isolates.

The tree topology based on the nt-concatamer was the same irrespective of using RAxML (Figure 4) or IQ-tree (not shown), and was consistent with the known phylogenetic reconstruction of VHSV based on the G gene. Genotypes I and III are monophyletic groups with 100% bootstrap support (BS) (Figure 4), as well as subtypes Ia, Ib, Ic, Id, and Ie (with 97 to 100% BS). With the exception of subtype Ie being the sister of other genotype I clusters (BS = 100%), phylogenetic relationships among subtypes of genotype I are difficult to identify, as they seem to be the result of a rapid radiation with very short branches and low BS. The only strongly supported relationship was the placement of Ib and Id as sister groups (BS > 90%). Ia and Ic were also recovered as sister groups but with low BS (<50%) forming a clade with the oldest VHSV isolate known (i.e., F1), although this relationship also showed low support (BS < 50%).

The phylogenetic tree shown in Figure 4 was annotated labeling taxa with a color code that identified different virulence classes established as above, where red was “H” (CPM > 42%), orange was “M” (14% < CPM ≤ 42%) and blue was “L” (CPM ≤ 14%). Notably, virulence in rainbow trout showed a strong phylogenetical component, except for “M” strains which were interspersed along the tree topology with no apparent pattern. The tree shows for each taxon the aa signatures associated with virulence (i.e., virulence haplotypes). Specific signatures were labeled with a color code to ease data visualization (red: aa significantly associated with the “H” phenotype; blue: aa significantly associated with the “L” phenotype; white: aa with no significant association). In general, SAPs-virulence appeared conserved among viruses belonging to the same virulence class, but we could observe several exceptions to this pattern. Subtype Ia consisted almost entirely of highly virulent isolates, apart from isolate VHSV/O.mykiss/I/TN/480/Oct96 (“M”; CPM = 14.5%) for which it was not possible to identify aa signatures that might explain its phenotype. Indeed, VHSV/O.mykiss/I/TN/480/Oct96 harbored Q and R residues at positions G212 and L298 which were unique to this strain. However, such signatures were not associated with any of the “H” or “L” groups, most likely due to limitations of our dataset; therefore, this observation should not be considered conclusive. Another exception is isolate DK-3612 (Ic), harboring an E at position N46, with no significant association with the “H” nor to the “L” categories. Similarly, Trabzon 207111 (Ie) showed a milder virulence phenotype in comparison to strains GE 1.2 and 2009-50-315-1 of the same subtype, but no relevant differences in SAPs-virulence were observed when compared to other “H” and “M” isolates within the same subtype. Viruses classified as Id, I, and IVa, encompassing only low virulent and one moderate strains, showed mixed “L” and “H” signatures, and none of the identified SAPs accounted for their diverse phenotypes. Similarly, strain M.rhabdo, a low virulent isolate within the Ib subtype, showed nine signatures significantly associated with the “H” phenotype, while causing no mortality. Finally, genotype III comprised two “L” and one “H” isolates. Differently from its close relatives, this latter isolate harbored highly virulent signatures at positions N393 and L149, which might determine its phenotype.

## Discussion

Several studies have attempted to link VHSV virulence to specific molecular markers based on phenotype information and/or genetic data (Betts and Stone, 2000; Campbell et al., 2009; Dale et al., 2009; Einer-Jensen et al., 2014; Kim et al., 2014; Ito et al., 2016, 2018; Baillon et al., 2017; Vakharia et al., 2019; Yusuff et al., 2019). However, because most of these attempts have been based on the visual inspection of a restricted number of sequences, the mechanisms underlying virulence are still elusive and lack statistical support. The scarce availability of whole genome sequences data represents a major constraint to the discovery of VHSV virulence markers. This limit is currently being overcome by the advent of next generation sequencing (NGS). Equally crucial to the identification of virulence markers is the use of empirical research that implements *in vitro*/*in vivo* models to identify genome regions modulating viral phenotype. While the use of these two strategies alone might be insufficient because of their inherent limitations, their combination appears effective in pinpointing mutations and/or motifs involved in virulence (Geoghegan and Holmes, 2018). In our study, we adopted this approach and integrated the use of *in vivo* models with whole genome sequencing to identify putative virulence markers in VHSV coding regions. To the best of our knowledge, this is the first study based on such an extensive dataset of experimental records and sequence data that encompasses the highest possible known variability in VHSV phenotypes and genotypes. This, combined with a robust statistical methodology to associate CPM scores with genetic polymorphisms, allowed the identification of putative virulence markers within coding regions at an unprecedented resolution.

One of the major challenges in this study was to classify the VHSV isolates into virulence classes to assist the identification of aa polymorphisms (SAPs) as putative virulence markers in rainbow trout. While this classification was a requirement for the approach herein used, it is not a straight-forward process to decide how the different phenotype categories should be determined. Here, a consortium of laboratories joined in a common effort to generate and combine cumulative mortality and sequence data. While the comparability of the genomes produced at different facilities was assessed through an intra-consortium proficiency test (Zamperin et al., 2019), *in vivo* virulence data could not undergo the same validation process. Some of the trials were conducted over a time span of several years with different positive control viruses, thus it was not possible to conduct a normalization of CPM among facilities. On the other hand, the concerted action of different laboratories allowed collecting mortality data for 55 VHSV isolates. The size of this dataset and the statistical methodology adopted was intended to mitigate, to a large extent, the effect of potential bias in the study.

The “H” and “L” categories described herein encompassed viruses that, according to our data, were homogeneous in terms of phenotype (as assessed by CPM values) and protein sequences. Less well defined was the establishment of the moderate virulence class, which was arbitrarily formed by genetically heterogeneous isolates that were not associated with either low or high virulence classes. For this reason, results associated with this group might change when subject to greater sampling. Indeed, as part of the Novimark project, with another set of experiments focused on determining the steps in the viral replication cycle involved in virulence (López-Vázquez et al., unpublished), we observed that the boundary between CPM and virulence classes was not always as mathematically clean as indicated here. An example is strain VHSV/O.mykiss/I/TN/480/Oct96, classified as “M” based on the criteria of the present study (CPM 14.5%), but showing a replication phenotype *in vitro* typical of “L” strains. A relevant issue when defining the *in vivo* virulence phenotype is that mortality can be influenced by many factors such as water temperature, fish genetics and size, housing density, homogeneity of viral stock, viral propagation and number of passages in cell culture, as well as inherent variations from tank to tank (Chevassus and Dorson, 1990; Lorenzen et al., 1999c; Skall et al., 2004; Hawley and Garver, 2008; Polverino et al., 2016). Consequently, a virulence phenotype described by CPM is subject to fluctuations depending on the experimental setting specific to each trial. Therefore, the cut off thresholds used for different virulence classes (CPM 42% and 14%) should be taken as a descriptor of our dataset, as CPM values from other *in vivo* trials with the same isolates might differ from those reported in this study. Consequently, the translation of these thresholds to fish trials performed under different conditions must be cautious.

In our study, we identified an extensive list of polymorphic sites (SAPs) interspersed throughout VHSV coding regions that varied significantly with virulence as well as aa signatures specifically associated with the high and low virulent phenotypes. Clearly, their effect on the virulence to rainbow trout must be validated under experimental conditions. The putative virulence markers identified also include SAPs located in the N and P protein coding regions, which have recently been suggested to be major virulence determinants for VHSV in rainbow trout (Ito et al., 2016, 2018; Vakharia et al., 2019). On the other hand, our results are not fully in line with previous works that have ruled out the role of the glycoprotein, the non-virion protein and of the polymerase in determining VHSV virulence in rainbow trout. In fact, Einer-Jensen et al. (2014) showed that the replacement of the G and NV genes of the Infectious Hematopoietic Necrosis Virus (IHNV) with their VHSV homologs, deriving either from high (Ia) or low (Ib) virulent strains in rainbow trout, had no significant impact on the survival rate of rainbow trout fingerlings. This was in accordance with a more recent study where the G and NV genes alone, or in the G-NV and G-NV-L combinations, were interchanged between low (IVb) and high virulent (Ia) VHSV clones, showing that the virulence phenotype of chimeric viruses was not governed by the donor genotype of G, NV, and L genes (Yusuff et al., 2019). However, these studies do not completely rule out the presence of virulence determinants in the G, NV and L genes, but indicate that, if present, they may need to interact with additional determinants elsewhere in the genome. This is consistent with previous studies where VHSV clones whose NV gene had been knocked-out showed reduced mortality in yellow perch and trout, indicating that this gene is needed for *in vivo* pathogenicity (Biacchesi et al., 2010; Ammayappan et al., 2011). In fact, it has been shown that a change from Arginine (R) to Serine (S) at position 116 of the NV protein is enough for attenuating VHSV virulence in rainbow trout experimentally challenged by immersion (Baillon et al., 2017). Interestingly, this amino acid change was identified also in our analysis, where aa signatures R and S were significantly associated with the high and low virulent phenotype, respectively. Similarly, a recent study using a chimeric VHSV generated by reverse genetics suggested that positions N46, N82, N83, and P39, also identified in our study, were putative markers of virulence (Vakharia et al., 2019), highlighting once again the potential of the analytical model herein presented for predicting virulence determinants. Indeed, reverse genetics is the most appropriate tool to verify the SAPs identified in this work. However, studies carried out so far using this methodology are mostly based on chimeric constructs where entire genes are replaced with their homolog counterpart of another ancestry (Einer-Jensen et al., 2014; Vakharia et al., 2019; Yusuff et al., 2019). By contrast, *in vivo* testing of clones subject to site-directed mutagenesis is scarce. Kim et al. (2014) tested the effect of specific amino acid signatures on *in vitro* virulence at some of the positions identified within this study, namely NV57, NV80, L149, and L298, but no change in the ability of the recombinant virus to infect and replicate in rainbow trout gill epithelial cells was found. Moreover, this study found evidence that a single amino acid change in the polymerase gene (I1012F) was enough to change virulence *in vitro* using rainbow trout epithelial cells. It is not clear, however, how well these results can be correlated to *in vivo* studies, as a thorough evaluation of the usefulness of rainbow trout gill epithelial cells as a proxy for virulence in fish is lacking. So far, the correlation between VHSV virulence *in vivo* and *in vitro* has only been assessed by Brudeseth et al. (2008) who demonstrated that a high virulent isolate infected and caused cytotoxic effect in gill epithelial cells faster than a low virulent, marine derived isolate. Further studies using a wide range of VHSV isolates would be necessary to clarify and understand the differences among high and low virulence VHSV isolates in cell culture. The same remark could be made on studies investigating the effect of VHSV genes as virulence markers and challenging the trout by intra-peritoneal injection (Vakharia et al., 2019; Yusuff et al., 2019). It has been demonstrated that fins are the main portal of entry in trout of IHNV, a closely related virus, and that a NV knockout virus was blocked at this site, which explains in part its low pathogenicity (Harmache et al., 2006). Thus, there is a risk by injection of bypassing this natural infection barrier and excluding some important virulence markers essential in the entry and early spread of VHSV in its host.

The phylogenetic reconstruction of viral coding regions, coupled with *in vivo* phenotype information, clearly indicate that VHSV virulence is largely determined by viral genetic make-up. As all but one of the isolates included in genotype Ia are highly virulent, we may assume that many of the signatures associated to high virulence have originated on the branch leading to this clade. Although this seems true for many of the SAPs, particularly within the G-gene, for at least 12 of the identified polymorphisms the virulent variant seems to be the ancestral state in the phylogeny of VHSV, with the “H” variant present in both high and low virulent isolates (Figure 4). Indeed, only genotype Ib seems completely devoid of high virulence variants, except for the isolate M.rhabdo. Very few parallelisms in the way of clear-cut shared convergent mutations were found among highly virulent isolates through the whole phylogeny of VHSV. This may indicate that virulence in rainbow trout was not achieved in the same way in different genotypes and subtypes. Obviously, it is clear that our data proved insufficient to entirely describe the genotype-phenotype associations of the viruses in our dataset, as some of the phenotypes could not be explained by the aa signatures observed at the SAPs-virulence herein identified. In truth, some of the polymorphisms might not necessarily be determinants of virulence, but rather the result of a natural evolutionary process of VHSV. One obvious example is VHSV/O.mykiss/I/TN/480/Oct96, the lone Ia isolate with considerably reduced virulence in rainbow trout (Baillon et al., 2017). The only virulence markers in this isolate different from other Ia isolates were G212 (Q), and L298 (R). As both signatures were unique to this isolate, no statistical association with virulence was possible. Another strategy to investigate the reduced virulence of this isolate would be to compare it at sites other than SAPs-virulence, with relatives sharing the same ancestry but with contrasting phenotypic differences. When doing so, we found 15 additional aa differences with close relatives from subtype Ia. Indeed, there is evidence that one amino acid change from A to E in position 241 of the N protein may be involved in the reduction of virulence for this isolate. Similarly, genotype III isolate NO-2007-50-385 from rainbow trout (“H”) differs in only two of the SAPs-virulence from its lower virulence sister isolate DK-4p101 (N393 and L149). It is surprising to notice that only two SAPs may be responsible for such dramatic changes in virulence (0 to 69% CPM), indicating that we should be looking for genotype and subtype specific virulence determinants, as suggested previously (Campbell et al., 2009; Ito et al., 2016, 2018). Indeed, changes in N-T118A, N-D121N, and N-N123S were claimed to be responsible for shift of virulence to rainbow trout in genotype III (Ito et al., 2016), but they were not identified herein as virulence SAPs, possibly due to the few isolates of this genotype included in our dataset.

In our study, we also attempted to understand whether VHSV virulence might be related to adaptive mechanisms of the virus to the host, and in particular to rainbow trout. Initially, as many as 21 polymorphisms appeared to be related to both the variables “virulence” and “host tropism.” However, when the strength of the association was assessed including both variables in the multinomial regression models, only SAPs N46, N401, and L149 appeared significantly associated with both traits. This evidence leads us to speculate that these markers might be host tropism determinants and might act as virulence markers in a host-dependent manner. In this view, the study of these SAPs and their implication in virulence in terms of viral-host interactome appears particularly interesting. Differently, the remaining 35 SAPs resulted solely associated with virulence. Although such observation seems to be contradictory, it can be explained with the moderate co-dependence of the variables “virulence” and “host tropism,” with the former having the highest predictive strength.

One of the limitations of our work is that we were not able to provide any information on co-occurring mutations. This is relevant in light of the recent study by Vakharia et al. (2019) that hypothesizes a concerted action of the N and P proteins in VHSV virulence. In future studies, the frequencies observed for the aa signatures for SAPs-virulence could be one possible criterion to detect SAPs that are subject to genetic linkage. Indeed, SAPs showing the same frequency scores are more likely to be co-occurring, either when they are located within the same gene in order to produce a more stable and functional protein, in genes that encode for proteins that interact with each other (e.g., N and P), or in proteins that interact with specific RNA motifs (e.g., the RNP complex and the genome RNA). Another consideration that must be addressed is that virulence markers were investigated in the current study only in coding regions. Notably, it has been shown that a single substitution in the 3′-terminus of VHSV directly affects growth kinetics *in vitro* (Kim et al., 2015), but the role of the different intergenic regions, especially in *in vivo* studies, has gone completely unexplored in the literature so far.

To date, the molecular basis for VHSV virulence in rainbow trout is still largely undetermined because of the scarce availability of sequence and *in vivo* data. Within this study, we have generated the largest dataset of *in vivo* virulence records and complete VHSV genomes and correlated phenotype and genetic data applying statistical methods. This experimental strategy allowed us to identify 38 putative virulence markers for VHSV virulence in rainbow trout and to provide for the first time a wide and representative list of candidate virulence markers for their subsequent confirmation. With the availability of laboratories facilities for reverse genetics, the data presented here offer a challenging opportunity to explore VHSV virulence and verify the role of specific signatures in viral ecology, including replication, infectivity, pathogenicity, transmissibility and tropism. A selection of the most promising markers herein identified were investigated through the generation of recombinant viruses, and their implications in *in vivo* virulence using rainbow trout as a model are presented in a linked manuscript (Baillon et al., submitted).

## Data Availability Statement

The sequence datasets presented in this study can be found in online repositories. The names of the repositories and accession numbers can be found in the Supplementary Material.

## Ethics Statement

The animal study was reviewed and approved by the Ministère de l’Éducation Nationale, de l’Enseignement Supérieur et de la Recherche, the Danish Animal Research Authority, and the Italian Ministry of Health.

## Author Contributions

VP, AC, SB, MB, and NO conceived the study. VP, AC, MG, SB, and NO developed the experimental design. AA, YB, DR, and MA propagated and sequenced viral isolates. AA, FP, TM, JC, LL, and AT performed experimental trials. VP, AC, MA, MG, and AA curated the datasets. MG carried out statistical analyses. AC performed the phylogenetic reconstruction. VP, AC, MG, CD, SB, AT, and NO interpreted and analyzed data. VP, AC, MG, CD, and SB drafted the manuscript. MG and MA performed graphics editing. All authors have read and approved the final manuscript.

## Conflict of Interest

The authors declare that the research was conducted in the absence of any commercial or financial relationships that could be construed as a potential conflict of interest.

## Footnotes

1 https://www.geneious.com

2 www.ncbi.nlm.nih.gov/Traces/sra/

3 https://www.ncbi.nlm.nih.gov/genbank/

4 http://www.fishpathogens.eu/
