# Supplementary material for: VHSV Single Amino Acid Polymorphisms (SAPs) Associated With Virulence in Rainbow Trout
Source: Front Microbiol. 2020 Aug 27;11:1984. doi: 10.3389/fmicb.2020.01984 (PMC7493562; doi:10.3389/fmicb.2020.01984)
Supplement: Supplementary file 2 [file Image_2.PDF]

# *Supplementary Material*

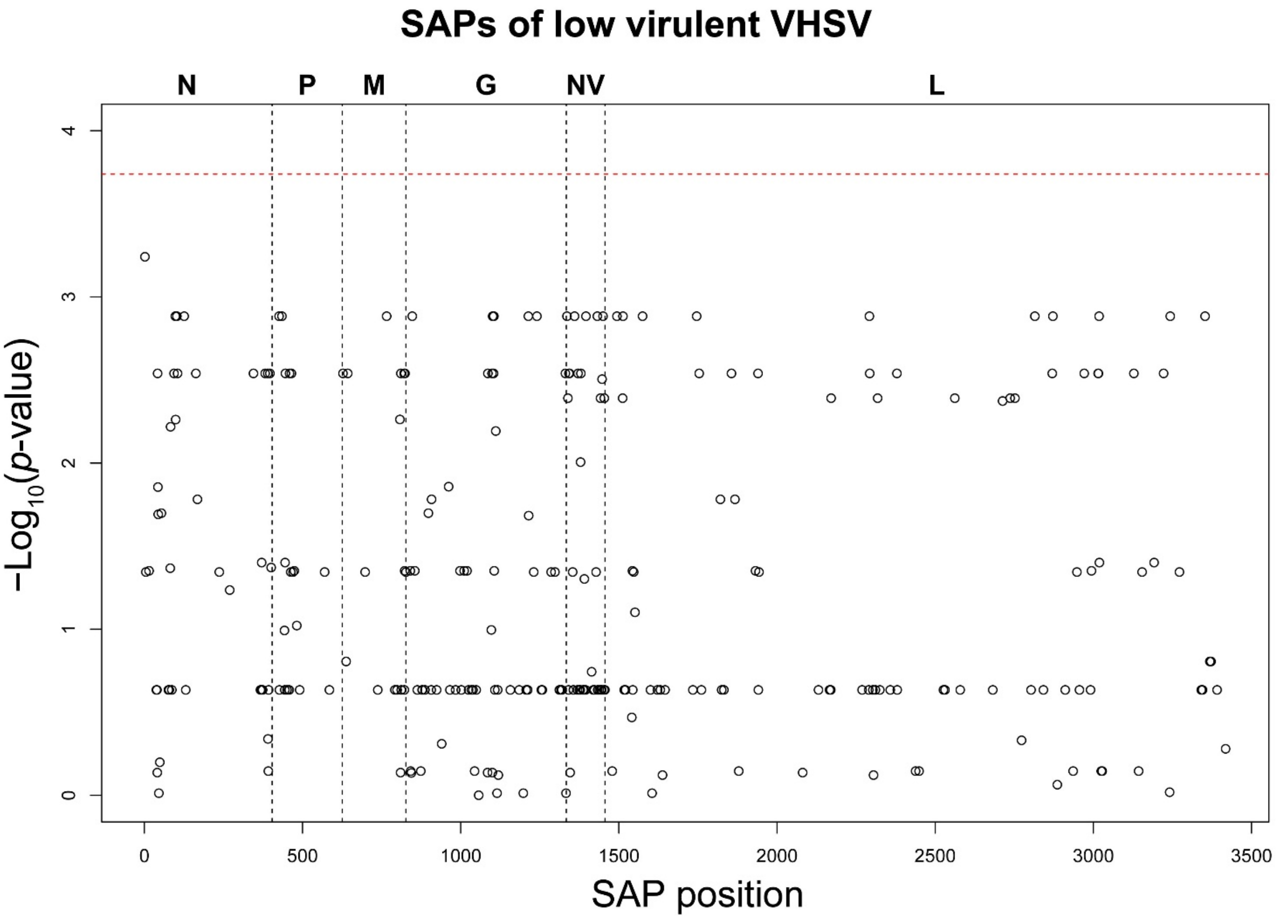


**Supplementary Figure 2. Manhattan plot of the association analysis conducted on low virulent isolates SAPs and the trait "virulence".** The SAP position along the genome coding regions is displayed on the *x*-axis, while the *y*-axis reports the negative logarithm of the association *p*-value. The red dashed line identifies the association test significance limit. As shown, no SAP was significant associated with virulence in this dataset.
