# Supplementary material for: VHSV Single Amino Acid Polymorphisms (SAPs) Associated With Virulence in Rainbow Trout
Source: Front Microbiol. 2020 Aug 27;11:1984. doi: 10.3389/fmicb.2020.01984 (PMC7493562; doi:10.3389/fmicb.2020.01984)
Supplement: Supplementary file 3 [file Image_3.PDF]

Supplementary Material

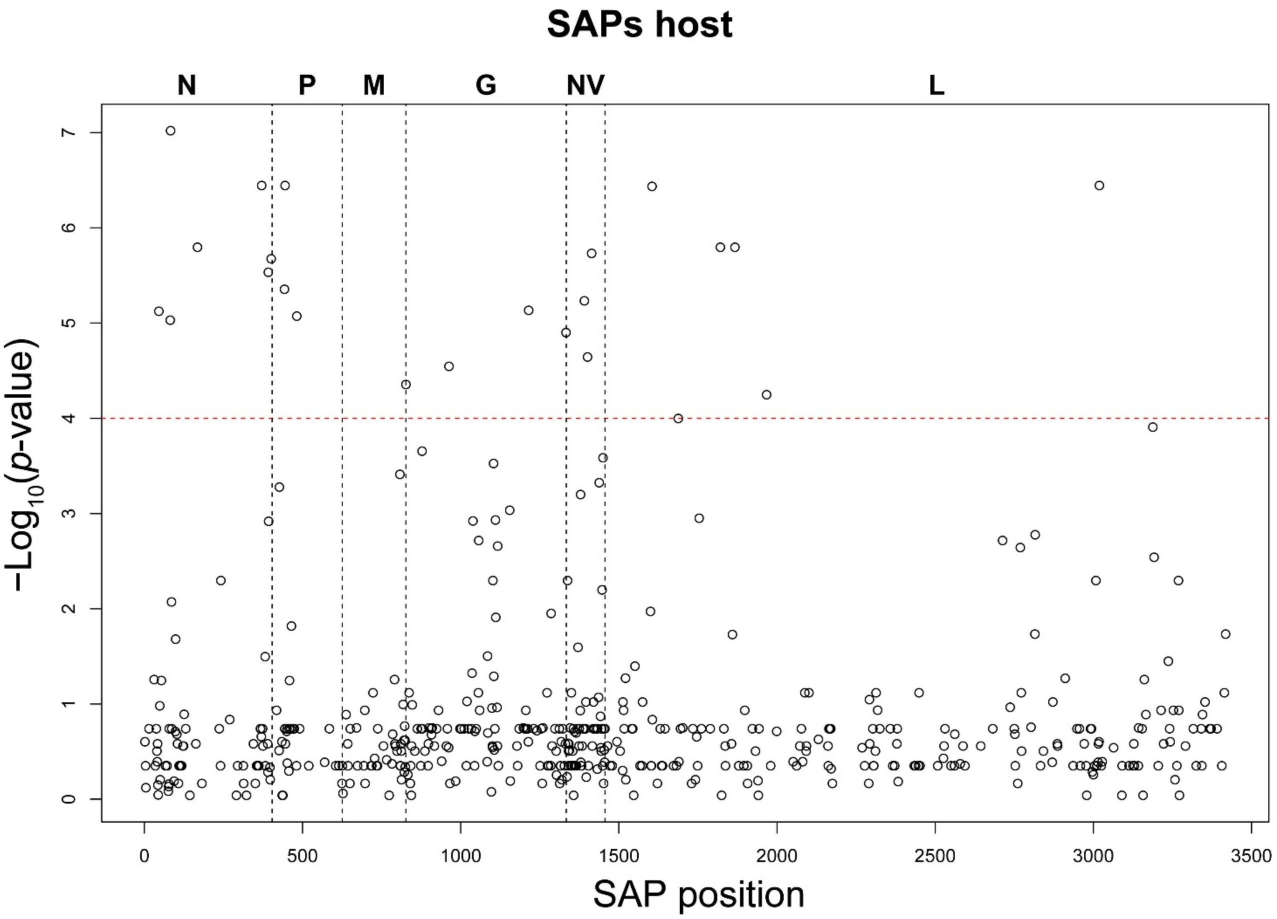

Supplementary Figure 3. Manhattan plot of the association analysis conducted VHSV SAPs and the trait “Host” referring to rainbow trout or other. The SAP position along the genome coding regions is displayed on the *x*-axis, while the *y*-axis reports the negative logarithm of the association *p*-value. The red dashed line identifies the association test significance limit.
