# Supplementary material for: VHSV Single Amino Acid Polymorphisms (SAPs) Associated With Virulence in Rainbow Trout
Source: Front Microbiol. 2020 Aug 27;11:1984. doi: 10.3389/fmicb.2020.01984 (PMC7493562; doi:10.3389/fmicb.2020.01984)
Supplement: Supplementary file 4 [file Table_1.PDF]

Supplementary Material

Supplementary Table 1. VHSV isolates subject to whole genome sequencing. For each strain, the SRA (Sequence Read Archive; www.ncbi.nlm.nih.gov/Traces/sra/), GenBank (https://www.ncbi.nlm.nih.gov/genbank/), and fish pathogens accession numbers (http://www.fishpathogens.eu/) are reported.

Isolate	SRA	GenBank	FishPathogens
F1	SRR11235464	MT162452	FP.VHSV.35
He-70	SRR11235465	MT162451	FP.VHSV.10
FR07/71	SRR11235457	MT162443	FP.VHSV.248
23/75	SRR9108123	MN038343	FP.VHSV.306
FR02/84	SRR9108124	MN038333	FP.VHSV.247
1458	SRR11235472	MT162445	FP.VHSV.252
3771P	SRR11235471	MT162446	FP.VHSV.2180
N11298	SRR11235470	KY793102	FP.VHSV.496
MM73	SRR11235469	MT162447	FP.VHSV.2181
1236-01	SRR11235468	MT162448	FP.VHSV.2182
DK-6137	SRR9108120	MN038338	FP.VHSV.225
DK-3345	SRR8908924	MK829395	FP.VHSV.12
DK-3592B	SRR11235467	MT162449	FP.VHSV.13
DK-6435	SRR11235456	MT162444	FP.VHSV.276
DK-7054	SRR8908926	MK829411	FP.VHSV.277
DK-7300	SRR8908919	MK829412	FP.VHSV.29
DK-9895174	SRR8908922	MK829413	FP.VHSV.269
DK-203490	SRR11235466	MT162450	FP.VHSV.677
VHSV/O.mykiss/I/TN/480/Oct96	SRR8943877	MK829677	FP.VHSV.2183
VHSV/O.mykiss/I/PN/234/Mar99	SRR8943893	MK829675	FP.VHSV.2184
VHSV/O.mykiss/I/BZ/301/Jun00	SRR8943899	MK829678	FP.VHSV.2185
VHSV/O.mykiss/I/TV/3/Dec02	SRR8943898	MK829679	FP.VHSV.2186
VHSV/O.mykiss/I/TV/299/Aug04	SRR8943896	MK829681	FP.VHSV.2187
VHSV/O.mykiss/I/TN/475/Nov04	SRR8943897	MK829680	FP.VHSV.2188
VHSV/S.trutta/I/TN/470/Nov09	SRR8943894	MK829676	FP.VHSV.2189
VHSV/O.mykiss/I/TN/80/Mar10	SRR8943895	MK829682	FP.VHSV.2190
VHSV/O.mykiss/I/TN/28/Feb11	SRR8943881	MK829683	FP.VHSV.2191
VHSV/O.mykiss/I/TN/62/Feb15	SRR8943891	MK829684	FP.VHSV.2192
VHSV/O.mykiss/I/TN/68/Feb15	SRR8943892	MK829685	FP.VHSV.2193
VHSV/O.mykiss/I/TN/84/Feb15	SRR8943889	MK829686	FP.VHSV.2194
DK-1p8	SRR9108116	MN038341	FP.VHSV.52
M.rhabdo	SRR11235459	MT162441	FP.VHSV.36
DK-5p276	SRR11235458	MT162442	FP.VHSV.81
DK-5p405	SRR8908909	MK829405	FP.VHSV.83
DK-5p26	SRR8908914	MK829403	FP.VHSV.72
DK-5e454	SRR8908916	MK829399	FP.VHSV.84

DK-5p393	SRR8908912	MK829404	FP.VHSV.82
DK-5p508	SRR8908917	MK829407	FP.VHSV.91
DK-5p795	SRR8908923	MK829409	FP.VHSV.135
DK-5p263	SRR8908911	MK829402	FP.VHSV.80
DK-5p11	SRR8908913	MK829401	FP.VHSV.68
DK-5p457	SRR8908910	MK829406	FP.VHSV.90
DK-5p785	SRR8908918	MK829408	FP.VHSV.133
SE-SVA-1033-9C	SRR8908921	MK829415	FP.VHSV.1117
DK-2149	SRR11235461	MT162439	FP.VHSV.791
DK-3612	SRR11235460	MT162440	FP.VHSV.802
NO-A163-68-EG46	SRR9108126	MN038331	FP.VHSV.249
FiP02b.00	SRR9108133	MN038325	FP.VHSV.232
GE 1.2	SRR11235462	MT162438	FP.VHSV.234
Trabzon 207111	SRR9108134	MN038326	FP.VHSV.2195
2009-50-315-1	SRR11235463	MT162437	FP.VHSV.2196
FR-L59x	SRR9108124	MN038333	FP.VHSV.275
DK-4p101	SRR11235474	MT162435	FP.VHSV.66
NO-2007-50-385	SRR11235473	MT162436	FP.VHSV.250
JF00Ehi1	SRR9108132	MN038328	FP.VHSV.226
