# Supplementary material for: VHSV Single Amino Acid Polymorphisms (SAPs) Associated With Virulence in Rainbow Trout
Source: Front Microbiol. 2020 Aug 27;11:1984. doi: 10.3389/fmicb.2020.01984 (PMC7493562; doi:10.3389/fmicb.2020.01984)
Supplement: Supplementary file 5 [file Table_2.PDF]

Supplementary Material

Supplementary Table 2. Single amino acid polymorphisms (SAPs) associated to mortality variations. For each SAP, the *p*-value and the genome location are reported.

SAP	*p*-value	Gene
N46	1,70e-07	N
N82	1,01e-07	N
N83	1,50e-09	N
N86	2,69e-05	N
N168	6,30e-08	N
N371	1,86e-08	N
N392	3,80e-10	N
N393	3,19e-05	N
N401	2,29e-06	N
P23	1,27e-07	P
P39	4,35e-07	P
P41	1,86e-08	P
P78	1,25e-06	P
M182	1,29e-08	M
M201	1,32e-11	M
G51	1,33e-11	G
G136	3,89e-09	G
G212	4,28e-07	G
G258	6,43e-06	G
G277	2,19e-11	G
G283	5,85e-08	G
G290	1,30e-08	G
G328	1,22e-05	G
G388	3,32e-07	G
NV45	1,03e-10	NV
NV57	3,91e-07	NV
NV67	1,91e-11	NV
NV80	2,50e-08	NV

SAP	*p*-value	Gene
NV104	1,29e-10	NV
NV113	9,89e-09	NV
NV116	2,48e-11	NV
L149	9,46e-11	L
L232	6,26e-13	L
L298	4,37e-06	L
L365	6,30e-08	L
L411	6,30e-08	L
L511	1,81e-12	L
L1313	3,90e-11	L
L1360	1,77e-05	L
L1563	1,86e-08	L
L1732	1,66e-11	L
