# Supplementary material for: VHSV Single Amino Acid Polymorphisms (SAPs) Associated With Virulence in Rainbow Trout
Source: Front Microbiol. 2020 Aug 27;11:1984. doi: 10.3389/fmicb.2020.01984 (PMC7493562; doi:10.3389/fmicb.2020.01984)
Supplement: Supplementary file 6 [file Table_3.PDF]

Supplementary Material

Supplementary Table 3. Multi-trait association analysis. The test was carried out on SAPs that appeared associated to both “virulence” and “host tropism” traits. The significance of the association of each trait, conditionally to each other, was assessed by likelihood ratio test ($p < 0.05$).

SAP	Logistic regression	Method	Likelihood ratio test	
			Host tropism	Virulence
N46	multinomial	PML*	$X^2 = 10.2$, df = 3, $p = 1.7 \times 10^{-2}$	$X^2 = 15.4$, df = 6, $p = 1.8 \times 10^{-2}$
N82	binomial	PML*	$X^2 = -0.035$, df = 1, $p = 1$	$X^2 = 16.9$, df = 2, $p = 2.1 \times 10^{-4}$
N83	multinomial	PML*	$X^2 = 4.83$, df = 2, $p = 8.9 \times 10^{-2}$	$X^2 = 18.4$, df = 4, $p = 1.0 \times 10^{-3}$
N168	binomial	PML*	$X^2 = 1.71$, df = 1, $p = 0.19$	$X^2 = 12.3$, df = 2, $p = 2.1 \times 10^{-3}$
N371	binomial	PML*	$X^2 = 2.44$, df = 1, $p = 0.12$	$X^2 = 13.8$, df = 2, $p = 9.9 \times 10^{-4}$
N392	binomial	ML**	$X^2 = 3.47$, df = 1, $p = 6.2 \times 10^{-2}$	$X^2 = 21.1$, df = 2, $p = 2.6 \times 10^{-5}$
N401	binomial	ML**	$X^2 = 4.42$, df = 1, $p = 3.5 \times 10^{-2}$	$X^2 = 7.59$, df = 2, $p = 2.2 \times 10^{-2}$
P39	multinomial	PML*	$X^2 = 6.05$, df = 4, $p = 0.19$	$X^2 = 16.9$, df = 8, $p = 3.0 \times 10^{-2}$
P41	binomial	PML*	$X^2 = 2.44$, df = 1, $p = 0.12$	$X^2 = 13.8$, df = 2, $p = 9.9 \times 10^{-4}$
P78	binomial	ML**	$X^2 = 1.62$, df = 1, $p = 0.20$	$X^2 = 11.5$, df = 2, $p = 3.1 \times 10^{-3}$
M201	multinomial	PML*	$X^2 = 3.96$, df = 2, $p = 0.14$	$X^2 = 27.8$, df = 4, $p = 1.4 \times 10^{-5}$
G136	binomial	PML*	$X^2 = 2.15$, df = 1, $p = 0.14$	$X^2 = 22.3$, df = 2, $p = 1.4 \times 10^{-5}$
G388	multinomial	PML*	$X^2 = 2.71$, df = 2, $p = 0.26$	$X^2 = 12.6$, df = 4, $p = 1.3 \times 10^{-2}$
NV57	multinomial	PML*	$X^2 = 2.71$, df = 2, $p = 0.26$	$X^2 = 12.5$, df = 4, $p = 1.4 \times 10^{-2}$
NV67	binomial	PML*	$X^2 = 0.33$, df = 1, $p = 0.56$	$X^2 = 25.6$, df = 4, $p = 2.7 \times 10^{-6}$
NV80	multinomial	PML*	$X^2 = 2.73$, df = 2, $p = 0.26$	$X^2 = 25.6$, df = 4, $p = 2.7 \times 10^{-6}$
L149	binomial	PML*	$X^2 = 4.57$, df = 1, $p = 3.3 \times 10^{-2}$	$X^2 = 20.9$, df = 2, $p = 2.8 \times 10^{-5}$
L365	binomial	PML*	$X^2 = 1.71$, df = 1, $p = 0.19$	$X^2 = 12.3$, df = 2, $p = 2.1 \times 10^{-3}$
L411	binomial	PML*	$X^2 = 1.71$, df = 1, $p = 0.19$	$X^2 = 12.3$, df = 2, $p = 2.1 \times 10^{-3}$
L511	binomial	PML*	$X^2 = 0.33$, df = 1, $p = 0.56$	$X^2 = 29.2$, df = 2, $p = 4.4 \times 10^{-7}$
L1563	binomial	PML*	$X^2 = 2.44$, df = 1, $p = 0.12$	$X^2 = 13.8$, df = 2, $p = 9.9 \times 10^{-4}$

* Penalized Maximum Likelihood

** Maximum Likelihood
