# Supplementary material for: VHSV Single Amino Acid Polymorphisms (SAPs) Associated With Virulence in Rainbow Trout
Source: Front Microbiol. 2020 Aug 27;11:1984. doi: 10.3389/fmicb.2020.01984 (PMC7493562; doi:10.3389/fmicb.2020.01984)
Supplement: Supplementary file 7 [file Table_4.PDF]

Supplementary Material

Supplementary Table 4. Amino acid signatures associated to different phenotypes (high and low virulent). For each "SAP-virulence", all the significant polymorphisms observed and the respective *p*-value (< 0.05) are reported.

SAP virulence	Variants	Virulence: Low	Virulence: High
N46	G	2.75e-04	
	K		7.94e-03
	R		2.31e-08
N82	E		1.4e-08
	G	8.55e-07	
N83	M	4.99e-08	
	T		5.92e-09
N168	H		5.1e-08
	Y	1.92e-07	
N371	K		7.55e-09
	R	4.99e-08	
N392	E	2.55e-05	
	G		9.34e-12
N393	E		3.65e-08
N401	E		3.76e-08
	G	8.6e-05	
P23	K	2.26e-03	
	R		3.38e-10
P39	P	4.99e-08	
	T		1.29e-08
P41	E	4.99e-08	
	G		7.55e-09
P78	F		4.62e-09
	L	2.97e-05	
M182	I		1.27e-04
	M		1.55e-03

		Virulence	
SAP virulence	Variants	Low	High
	T	2.91e-06	
M201	R	1.78e-05	
	W		3.76e-11
G51	D		2.05e-13
	E	1.86e-04	
G136	D		2.73e-12
	N	1.24e-03	
G212	E		7.94e-03
	K		5.82e-09
	T	2.26e-03	
G277	A	3.55e-04	
	T		2.05e-13
G283	K	3.3e-03	
	N		4.61e-10
G290	I	7.43e-03	
	V		3.55e-11
G328	V		7.16e-08
G388	D		2.62e-07
	N	1.92e-07	
NV45	M	2.55e-03	
	V		2.05e-13
NV57	D		3.58e-07
	N	1.92e-07	
NV67	H		1.1e-11
	Y	5.93e-05	
NV80	G		2.97e-05
	K	4.99e-08	
	R		8.41e-04
NV104	I	7.74e-04	
	V		7.5e-13
NV113	I	1.94e-03	
	L		2.87e-12

		Virulence	
SAP virulence	Variants	Low	High
NV116	R		2.05e-13
	S	8.41e-04	
L149	E		4.3e-11
	G	3.36e-06	
L232	I		3.22e-12
	V	9.55e-06	
L298	E	1.71e-03	
	K		3.76e-11
L365	I		5.1e-08
	V	1.92e-07	
L411	F		5.1e-08
	Y	1.92e-07	
L511	K	7.67e-05	
	R		2.05e-13
L1313	M		4.61e-10
	T	9.55e-06	
	V		7.94e-03
L1563	I	4.99e-08	
	L		7.55e-09
L1732	A		2.05e-13
	T	5.93e-05	
